# Spetzler-Martin grade I and II cerebral arteriovenous malformations: a propensity-score matched analysis of resection and stereotactic radiosurgery in adult patients

**DOI:** 10.1007/s10143-025-03431-2

**Published:** 2025-02-28

**Authors:** Salem M. Tos, Mahmoud Osama, Georgios Mantziaris, Bardia Hajikarimloo, Nimer Adeeb, Sandeep Kandregula, Adam A. Dmytriw, Hamza Adel Salim, Basel Musmar, Kareem El Naamani, Christopher Ogilvy, Douglas Kondziolka, Ahmed Abdelsalam, Deepak Kumbhare, Sanjeev Gummadi, Cagdas Ataoglu, Ufuk Erginoglu, Muhammed Amir Essibayi, Abdullah Keles, Sandeep Muram, Daniel Sconzo, Howard Riina, Arwin Rezai, Johannes Pöppe, Rajeev D. Sen, Omar Alwakaa, Christoph J. Griessenauer, Pascal Jabbour, Stavropoula I. Tjoumakaris, Jan-Karl Burkhardt, Robert M. Starke, Mustafa Baskaya, Laligam N. Sekhar, Michael R. Levitt, David J. Altschul, Neil Haranhalli, Malia McAvoy, Assala Aslan, Abdallah Abushehab, Christian Swaid, Adib Abla, Christopher Stapleton, Matthew Koch, Visish M. Srinivasan, Peng R. Chen, Spiros Blackburn, Mark J. Dannenbaum, Omar Choudhri, Bryan Pukenas, Darren Orbach, Edward Smith, Markus Möhlenbruch, Ali Alaraj, Ali Aziz-Sultan, Aman B. Patel, Hugo H. Cuellar, Michael Lawton, Jacques Morcos, Bharat Guthikonda, Jason Sheehan

**Affiliations:** 1 Department of Neurosurgery, University of Virginia, Charlottesville, VA USA; 2 Department of Neurosurgery, Louisiana State University Health Science Center, Shreveport, LA USA; 3 Department of Neurosurgery, McGovern Medical School, UT Health Sciences Center at Houston, Houston, TX USA; 4 Neuroendovascular Program, Massachusetts General Hospital, Harvard Medical School, Boston, MA USA; 5 Department of Radiology, Louisiana State University, Shreveport, LA USA; 6 Department of Neurosurgery, Thomas Jefferson University Hospital, Philadelphia, PA USA; 7 Division of Neurosurgery, Beth Israel Deaconess Medical Center, Harvard Medical School, Boston, MA USA; 8 Department of Neurosurgery, New York University Grossman School of Medicine, New York, NY USA; 9 Department of Neurosurgery, University of Miami, Miller School of Medicine, Miami, FL USA; 10 Department of Neurosurgery, University of Wisconsin School of Medicine, Madison, WI USA; 11 Montefiore Einstein Cerebrovascular Research Laband, Department of Neurological Surgery, Montefiore Medical Centeraq, Albert Einstein College of Medicine, New York, NY USA; 12 Department of Neurosurgery, Paracelsus Medical University, Christian Doppler Klinik, Salzburg, Austria; 13 Department of Neurosurgery, University of Washington, Seattle, WA USA; 14 Department of Plastic Surgery, Mayo Clinic Hospital, Rochester, MN USA; 15 Department of Neurosurgery, University of Florida, Gainesville, FL USA; 16 Neurointerventional Radiology, Boston Children’s Hospital, Harvard Medical School, Boston, MA USA; 17 Department of Neurosurgery, Boston Children’s Hospital, Harvard Medical School, Boston, MA USA; 18 Interventional Neuroradiology, Department of Neuroradiology, Heidelberg University Hospital, Heidelberg, Germany; 19 Department of Neurosurgery, University of Illinoisinaq, Chicago, IL USA; 20 Department of Neurosurgery, Brigham and Women Hospital, Harvard Medical School, Boston, MA USA; 21 Department of Neurosurgery, Barrow, Neurological Institute, Phoenix, AZ USA

**Keywords:** Cerebral arteriovenous malformations, Spetzler-Martin grade I and II, Resection, Stereotactic radiosurgery, AVM obliteration, Complication rates, Functional outcomes

## Abstract

Cerebral arteriovenous malformations (AVMs) are congenital vascular anomalies that can lead to severe complications, including hemorrhage and neurological deficits. This study compares the outcomes of microsurgical resection and stereotactic radiosurgery (SRS) for SM grade I and II AVMs. Out of a large multicenter registry, we identified 180 matched patients with SM grade I and II AVMs treated with either microsurgical resection or SRS between 2010 and 2023. The primary outcomes were AVM obliteration rates and complications; secondary outcomes included neurological status and functional outcomes measured by the modified Rankin Scale (mRS). Propensity score matching (PSM) was utilized to ensure comparability between treatment groups. After PSM, 90 patients were allocated to each treatment group. Significant differences were observed in complete obliteration rates, with resection achieving higher rates compared to SRS in overall cases (97.8% vs. 60.0%, p < 0.001), unruptured AVMs (100% vs. 58.3%, p < 0.001), and ruptured AVMs (95.2% vs. 61.9%, p < 0.001). Functional improvement rates were similar between the groups for overall cases (67.2% in resection vs. 66.7% in SRS, p = 0.95), unruptured AVMs (55.2% in resection vs. 55.6% in SRS, p > 0.9), and ruptured AVMs (78.1% in resection vs. 74.1% in SRS, p = 0.7). Symptomatic complication rates were identical between the groups (11.1% each, p > 0.9), while permanent complication rates were comparable (6.7% in resection vs. 5.6% in SRS, p = 0.8). Resection demonstrated significantly higher complete obliteration rates compared to SRS across all cases, including unruptured and ruptured AVMs. Functional improvement rates were similar between the two treatment groups, with no significant differences in symptomatic or permanent complication rates.

**Graphical Abstract:**

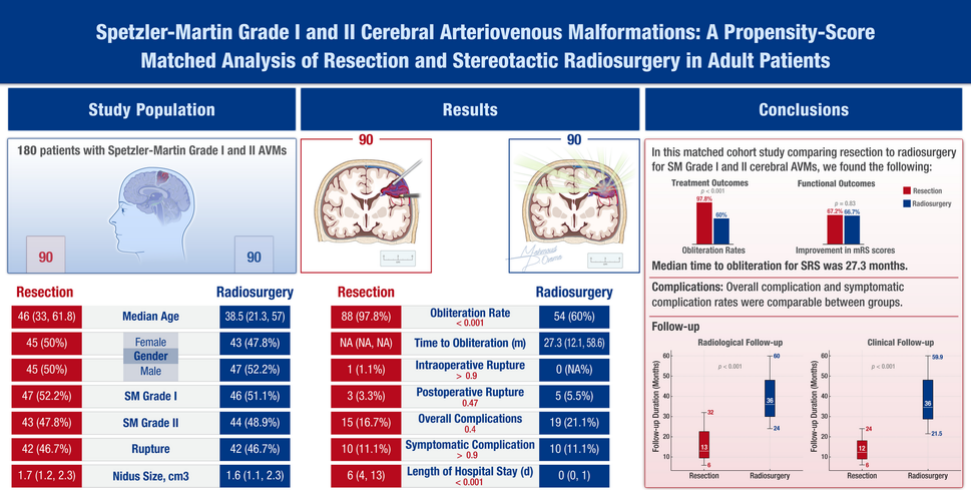

## Introduction

Cerebral arteriovenous malformations (AVMs) are congenital vascular anomalies characterized by abnormal connections between cerebral arteries and veins, bypassing the capillary system.[1] These high-flow lesions can lead to significant clinical complications, including intracranial hemorrhage, seizures, and neurological deficits.[2–4] The Spetzler-Martin (SM) grading system, which classifies AVMs based on size, location, and venous drainage patterns, is widely used to assess the risk of surgical intervention and guide treatment decisions.[5]SM grade I and II AVMs are considered low-grade lesions, typically associated with a more favorable prognosis and a higher likelihood of obliteration with appropriate treatment.[5]

The management of SM grade I and II AVMs has evolved significantly over the past few decades, with resection and stereotactic radiosurgery (SRS) emerging as the primary treatment modalities.[6, 7] Resection involves the direct surgical removal of the AVM nidus, offering the potential for immediate obliteration.[7] This approach, however, is invasive and carries risks of perioperative complications, including hemorrhage, infection, and new neurological deficits.[8] Despite these risks, resection has been shown to provide excellent long-term outcomes, particularly for small, superficially located AVMs that are easily accessible.[8, 9]

SRS, on the other hand, is a minimally invasive treatment that delivers high-dose focused radiation to the AVM nidus, inducing gradual obliteration of the lesion over time. SRS is particularly advantageous for deep-seated or eloquent AVMs that are challenging to access surgically.[6] The minimally invasive nature of SRS reduces the immediate risks associated with open surgery; however, the delayed obliteration and the potential for radiation-induced complications, such as radiation edema and hemorrhage during the latency period remain significant concerns.[6, 10]The optimal choice between resection and SRS often hinges on a careful consideration of patient-specific factors, including AVM characteristics, patient age, comorbidities, and the potential impact on quality of life.

Previous studies have extensively investigated the outcomes of resection and SRS for cerebral AVMs, with a particular focus on cure rates, complication profiles, and long-term functional outcomes. Given the evolving landscape of AVM management, our study aims to provide a contemporary comparative analysis of resection versus SRS for SM grade I and II AVMs.

## Methods

### Study design

This study was designed as a sub-analysis of the Multicenter International Study for Treatment of Brain AVMs (MISTA), which is a is a synthesis of consecutive brain AVMs treated by microsurgery, endovascular embolization, SRS, or combination of modalities at academic institutions in North America and Europe. The study aimed to compare outcomes of microsurgical resection versus SRS in SM grade I and II brain AVMs in adults treated between January 2010 and December 2023. Exclusion criteria included incomplete records, associated vascular malformations, prior treatments, combination treatments, and staged surgery or SRS. The study was approved by institutional review boards, with waived informed consent due to its retrospective design. Data was de-identified for confidentiality, and the STROBE guidelines were followed to ensure systematic and reproducible results.[11]

### Data source and variables

Collected data covered patient demographics, AVM characteristics, treatment specifics, and outcomes. Demographics included age, gender, and race. Clinical presentation involved symptoms like hemorrhage, seizure, headache, and neurological deficits. AVM characteristics included rupture status and timing, location, eloquence, nidus volume, SM grade, arterial feeders, draining veins, and presence of venous stenosis or associated aneurysms.

### Surgical modality, follow-up, and outcomes

**Fig. 1:**
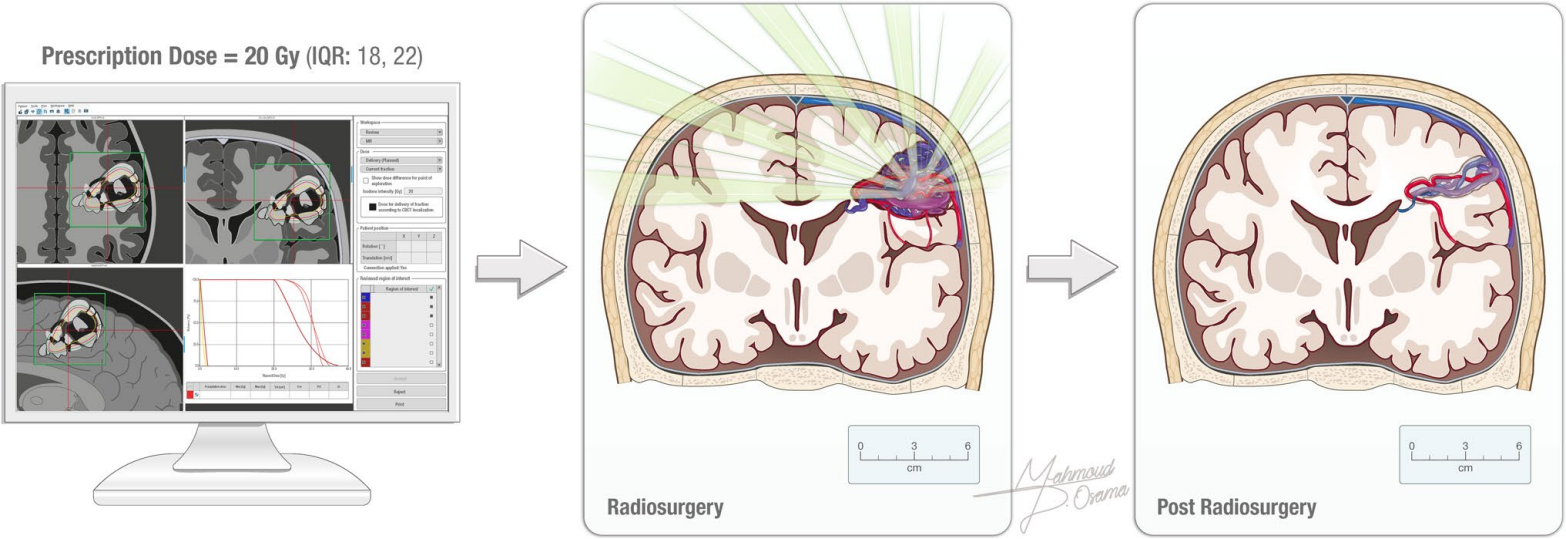
High-dose radiation beams are precisely targeted to the AVM, minimizing exposure to surrounding brain tissue and leading to gradual AVM obliteration

Treatment details varied by modality. Microsurgical resection patients were monitored postoperatively in a neurological unit, while SRS patients received Gamma Knife, CyberKnife®, or, linear accelerator (LINAC) treatments and were discharged the same day. The prescription dose was recorded (Fig. 1). Follow-up included clinical evaluations and imaging studies (MRI, MRA, CTA, and/or catheter angiography) at regular intervals at the discretion of the institution. Primary outcomes were AVM obliteration rates and safety, defined by complication rates and severity. Secondary outcomes included functional status at last follow-up, measured by the modified Rankin Scale (mRS), and other AVM-related symptoms.

### Statistical analysis

Statistical analysis was conducted using R (version 4.3.2, 2024; RStudio, Inc; R Foundation for Statistical Computing). Descriptive statistics for continuous variables were summarized as medians and interquartile ranges (IQR), and categorical variables as frequencies and percentages. Comparisons between resection and SRS cohorts were made using Wilcoxon rank-sum tests for continuous variables and Pearson's Chi-squared or Fisher's exact tests for categorical variables. Propensity score matching, performed with the "MatchIt" package using "optimal matching" method for the propensity score variables and “exact matching” for rupture status, eloquent or deep locations, ensured comparability between cohorts. Matching was based on SM grade, rupture status, nidus size, and eloquent or deep locations. A well-balanced match was indicated by a value < 0.10, with 0.10 to 0.20 signifying moderate balance. Time-to-event analyses were conducted using cumulative incidence curves to evaluate the rates of obliteration following SRS. Statistical significance was set at *p* < 0.05.

## Results

### Patient, AVM, and surgical treatment characteristics in the matched cohorts

The study included 281 patients (Resection: 148; SRS:133). After propensity score matching, 180 patients with SM grade I and II AVMs were analysed, with 90 patients in each cohort (Table 1). No significant differences were found in patient demographics between the cohorts. Presenting mRS score of 0 was more common in the SRS group (45.6% vs 23.3%, *p* = 0.016). Ruptured AVMs accounted for 46.7% of both groups. Resection was more common within 24 h of rupture (57.1% vs 23.7%), and SRS was more common > 14 days post-rupture (73.7% vs 19.0%, *p* < 0.001). Frontal AVMs were most common in both groups, but occipital AVMs were more frequent in the resection group (11.1% vs 3.3%, *p* = 0.013). The median nidus size was similar (1.7 cm^3^ vs 1.6 cm^3^, *p* = 0.5). The most common SRS modality was Gamma Knife (75.6%), followed by CyberKnife® (13.3%) and LINAC (11.1%), with a median prescription dose of 20.0 Gy (IQR: 18.0–22.0 Gy).

**Table 1:** Comparison of the patient, AVM, and treatment characteristics of the matched resection and stereotactic radiosurgery cohorts

Characteristic	Resection, N = 90^*1*^	Radiosurgery, N = 90^*1*^	*p*-value^*2*^
Age, year	46.0 (33.0, 61.8)	38.5 (21.3, 57.0)	0.054
Gender			0.8
Female	45 (50.0%)	43 (47.8%)	
Male	45 (50.0%)	47 (52.2%)	
Race			0.082
Asian	4 (5.1%)	1 (1.4%)	
Black	6 (7.6%)	16 (22.2%)	
Hispanic	7 (8.9%)	7 (9.7%)	
White	60 (75.9%)	46 (63.9%)	
Other	2 (2.5%)	2 (2.8%)	
Unknown	11	18	
Family History	2 (2.4%)	3 (4.1%)	0.7
Unknown	7	16	
Clinical presentation			
None	16 (17.8%)	23 (25.6%)	0.2
Hemorrhage	11 (12.2%)	5 (5.6%)	0.12
Headache	40 (44.4%)	35 (38.9%)	0.4
Seizure	12 (13.3%)	13 (14.4%)	0.8
Visual Disturbance	1 (1.1%)	6 (6.7%)	0.12
Speech deficits	1 (1.1%)	3 (3.3%)	0.6
Motor deficits	11 (12.2%)	11 (12.2%)	> 0.9
Confusion	9 (10.0%)	4 (4.4%)	0.15
Presentation mRS			0.016
0	21 (23.3%)	41 (45.6%)	
1	33 (36.7%)	26 (28.9%)	
2	21 (23.3%)	8 (8.9%)	
3	6 (6.7%)	6 (6.7%)	
4	2 (2.2%)	2 (2.2%)	
5	7 (7.8%)	7 (7.8%)	
Rupture	42 (46.7%)	42 (46.7%)	> 0.9
Rupture Timing			< 0.001
24 h	24 (57.1%)	9 (23.7%)	
< 7 days	5 (11.9%)	0 (0.0%)	
7–14 days	5 (11.9%)	1 (2.6%)	
> 14 days	8 (19.0%)	28 (73.7%)	
Unknown	0	4	
Location			
Frontal	23 (25.6%)	28 (31.1%)	0.4
Temporal	20 (22.2%)	21 (23.3%)	0.3
Parietal	15 (16.7%)	13 (14.4%)	0.8
Occipital	10 (11.1%)	3 (3.3%)	0.013
Cerebellar	19 (21.1%)	15 (16.7%)	> 0.9
Corpus Callosum	2 (2.2%)	0 (0.0%)	> 0.9
Insular	0 (0.0%)	2 (2.2%)	0.5
Thalamus	0 (0.0%)	2 (2.2%)	0.5
Basal Ganglia	1 (1.1%)	1 (1.1%)	0.6
Brainstem	0 (0.0%)	5 (5.6%)	0.059
Eloquent AVM location	29 (32.2%)	29 (32.2%)	> 0.9
Deep AVM location	20 (22.2%)	20 (22.2%)	> 0.9
Nidus Size, cm^3^	1.7 (1.2,2.3)	1.6 (1.1,2.3)	0.5
Spetzler-Martin Grade			0.9
I	47 (52.2%)	46 (51.1%)	
II	43 (47.8%)	44 (48.9%)	
Compacted	64 (72.7%)	64 (71.9%)	> 0.9
Unknown	2	1	
Number of Feeders			0.4
Multiple	54 (62.1%)	48 (55.8%)	
Single	33 (37.9%)	38 (44.2%)	
Unknown	3	4	
Number of Draining Veins			0.5
Multiple	23 (29.9%)	17 (24.6%)	
Single	54 (70.1%)	52 (75.4%)	
Unknown	13	21	
Location of Draining Veins			0.14
Both	6 (6.8%)	1 (1.1%)	
Deep	13 (14.8%)	17 (19.1%)	
Superficial	69 (78.4%)	71 (79.8%)	
Unknown	2	1	
Venous Stenosis	5 (6.9%)	10 (13.7%)	0.2
Unknown	18	17	
Nidal Aneurysm	9 (10.0%)	11 (12.2%)	0.6
SRS Modality			> 0.9
GKRS	0 (NA%)	68 (75.6%)	
Cyberknife	0 (NA%)	12 (13.3%)	
LINAC	0 (NA%)	10 (11.1%)	
Prescription dose, Gy	NA	20.0 (18.0,22.0)	
Length of hospital stay, days	6.0 (4.0,13.0)	0.0 (0.0,1.0)	< 0.001
Radiological follow-up, months	13.0 (6.0, 32.0)	36.0 (24.0, 60.0)	< 0.001
Clinical follow-up, months	12.0 (6.0, 24.0)	36.0 (21.5, 59.9)	< 0.001

^*1*^Median (IQR); n (%)^*2*^Wilcoxon rank sum test; Pearson's Chi-squared test; Fisher's exact test

Hospital stay was longer for the resection group (median 6.0 days [IQR 4–13]) compared to the SRS group (0 days [IQR 0–1], *p* < 0.001). Follow-up duration also differed, with the SRS group having longer radiological (36.0 [IQR 24.0, 60.0] months vs 13.0 [IQR 6.0, 32.0], *p* < 0.001) and clinical follow-ups (36.0 [IQR 21.5, 59.9] months vs 12.0 [IQR 6, 24.0], *p* < 0.001).

### Patient, AVM and surgical treatment characteristics: ruptured versus unruptured AVMs

We conducted subgroup analyses on the baseline characteristics of resection and SRS for both unruptured and ruptured AVMs (Table 2andTable 3). For unruptured AVMs (n = 96), there were no significant differences in age, gender, race, or presenting symptoms across the treatment groups, except for headaches, which were more frequent in the resection group (47.9% vs. 25.0%, p = 0.02). Presentation mRS scores differed significantly (*p* = 0.002), with more patients in the SRS group having mRS 0. AVM characteristics were similar between the cohorts but the duration of radiological (*p* = 0.002) and clinical (*p* < 0.001) follow-up was longer for SRS patients.

**Table 2:** Comparison of the patient, nidal, and treatment characteristics of the unruptured low-grade AVMs in the microsurgery and stereotactic radiosurgery cohorts

Characteristic	Resection, N = 48^*1*^	Radiosurgery, N = 48^*1*^	*p*-value^*2*^
Age, years	45.0 (33.0, 59.5)	36.0 (22.8, 54.3)	0.051
Gender			0.3
Female	28 (58.3%)	23 (47.9%)	
Male	20 (41.7%)	25 (52.1%)	
Race			0.11
Asian	2 (4.5%)	1 (2.6%)	
Black	1 (2.3%)	7 (17.9%)	
Hispanic	4 (9.1%)	5 (12.8%)	
White	35 (79.5%)	25 (64.1%)	
Other	2 (4.5%)	1 (2.6%)	
Unknown	4	9	
Family History	0 (0.0%)	3 (7.3%)	0.10
Unknown	3	7	
Clinical presentation			
None	13 (27.1%)	19 (39.6%)	0.2
Hemorrhage	1 (2.1%)	1 (2.1%)	> 0.9
Seizure	8 (16.7%)	10 (20.8%)	0.6
Headache	23 (47.9%)	12 (25.0%)	0.020
Visual Disturbance	1 (2.1%)	4 (8.3%)	0.4
Speech deficits	0 (0.0%)	0 (0.0%)	> 0.9
Motor deficits	7 (14.6%)	4 (8.3%)	0.3
Confusion	2 (4.2%)	1 (2.1%)	> 0.9
Presentation mRS			0.002
0	13 (27.1%)	30 (62.5%)	
1	25 (52.1%)	14 (29.2%)	
2	8 (16.7%)	2 (4.2%)	
3	1 (2.1%)	1 (2.1%)	
4	1 (2.1%)	0 (0.0%)	
5	0 (0.0%)	1 (2.1%)	
Location			0.8
Frontal	17 (35.4%)	16 (33.3%)	
Temporal	9 (18.8%)	11 (22.9%)	
Parietal	8 (16.7%)	7 (14.6%)	
Occipital	6 (12.5%)	3 (6.3%)	
Corpus Callosum	1 (2.1%)	0 (0.0%)	
Cerebellar	7 (14.6%)	7 (14.6%)	
Thalamus	0 (0.0%)	1 (2.1%)	
Basal Ganglia	0 (0.0%)	1 (2.1%)	
Brainstem	0 (0.0%)	2 (4.2%)	
Eloquent AVM location	11 (22.9%)	11 (22.9%)	> 0.9
Deep AVM location	6 (12.5%)	6 (12.5%)	> 0.9
Nidus Size, cm^3^	1.7 (1.3, 2.3)	1.7 (1.1, 2.4)	0.7
Spetzler-Martin Grade			> 0.9
I	33 (68.8%)	33 (68.8%)	
II	15 (31.3%)	15 (31.3%)	
Compacted	37 (78.7%)	38 (79.2%)	> 0.9
Unknown	1	0	
Number of Feeders			0.2
Multiple	33 (71.7%)	28 (59.6%)	
Single	13 (28.3%)	19 (40.4%)	
Unknown	2	1	
Number of Draining Veins			0.9
Multiple	15 (34.1%)	13 (32.5%)	
Single	29 (65.9%)	27 (67.5%)	
Unknown	4	8	
Location of Draining Veins			0.3
Both	3 (6.4%)	1 (2.1%)	
Deep	7 (14.9%)	4 (8.3%)	
Superficial	37 (78.7%)	43 (89.6%)	
Unknown	1	0	
Venous Stenosis	2 (5.0%)	6 (14.6%)	0.3
Unknown	8	7	
Nidal Aneurysm	3 (6.3%)	6 (12.5%)	0.5
Radiation Type			> 0.9
GKRS	0 (NA%)	36 (75.0%)	
Cyberknife	0 (NA%)	7 (14.6%)	
LINAC	0 (NA%)	5 (10.4%)	
Prescription dose, Gy	NA (NA, NA)	20.0 (18.0, 21.0)	
Length of hospital stay, days	5.0 (3.0,7.0)	0.0 (0.0,0.0)	< 0.001
Radiological follow-up, months	20.0 (6.0, 36.0)	36.0 (24.0, 69.0)	0.002
Clinical follow-up, months	13.0 (6.0, 25.0)	36.0 (23.2, 62.3)	< 0.001

^*1*^Median (IQR); n (%) ^*2*^Wilcoxon rank sum test; Pearson's Chi-squared test; Fisher's exact test

**Table 3:** Comparison of the patient, nidal, and treatment characteristics of the ruptured low-grade AVMs in the resection and stereotactic radiosurgery cohorts

Characteristic	Resection, N = 42^*1*^	Radiosurgery, N = 42^*1*^	*p*-value^*2*^
Age, years	48.0 (34.0, 64.3)	41.0 (18.8, 64.0)	0.5
Gender			0.5
Female	17 (40.5%)	20 (47.6%)	
Male	25 (59.5%)	22 (52.4%)	
Race			0.4
Asian	2 (5.7%)	0 (0.0%)	
Black	5 (14.3%)	9 (27.3%)	
Hispanic	3 (8.6%)	2 (6.1%)	
White	25 (71.4%)	21 (63.6%)	
Other	0 (0.0%)	1 (3.0%)	
Unknown	7	9	
Family History	2 (5.3%)	0 (0.0%)	0.5
Unknown	4	9	
Presentation			
None	3 (7.1%)	4 (9.5%)	> 0.9
Hemorrhage	10 (23.8%)	4 (9.5%)	0.079
Seizure	4 (9.5%)	3 (7.1%)	> 0.9
Headache	17 (40.5%)	23 (54.8%)	0.2
Visual Disturbance	0 (0.0%)	2 (4.8%)	0.5
Speech deficits	1 (2.4%)	3 (7.1%)	0.6
Motor deficits	4 (9.5%)	7 (16.7%)	0.3
Confusion	7 (16.7%)	3 (7.1%)	0.2
Presentation mRS			0.5
0	8 (19.0%)	11 (26.2%)	
1	8 (19.0%)	12 (28.6%)	
2	13 (31.0%)	6 (14.3%)	
3	5 (11.9%)	5 (11.9%)	
4	1 (2.4%)	2 (4.8%)	
5	7 (16.7%)	6 (14.3%)	
Rupture Timing			< 0.001
24 h	21 (53.8%)	9 (23.7%)	
< 7 days	5 (12.8%)	0 (0.0%)	
7–14 days	5 (12.8%)	1 (2.6%)	
> 14 days	8 (20.5%)	28 (73.7%)	
Unknown	3	4	
Location			0.064
Frontal	6 (14.3%)	12 (28.6%)	
Temporal	11 (26.2%)	10 (23.8%)	
Parietal	7 (16.7%)	6 (14.3%)	
Occipital	4 (9.5%)	0 (0.0%)	
Corpus Callosum	1 (2.4%)	0 (0.0%)	
Insular	0 (0.0%)	2 (4.8%)	
Cerebellar	12 (28.6%)	8 (19.0%)	
Thalamus	0 (0.0%)	1 (2.4%)	
Basal Ganglia	1 (2.4%)	0 (0.0%)	
Brainstem	0 (0.0%)	3 (7.1%)	
Eloquent AVM location	18 (42.9%)	18 (42.9%)	> 0.9
Deep AVM location	12 (28.6%)	12 (28.6%)	> 0.9
Nidus Size, cm^3^	1.9 (1.0, 2.2)	1.6 (1.0, 2.3)	0.6
Spetzler-Martin Grade			0.8
I	14 (33.3%)	13 (31.0%)	
II	28 (66.7%)	29 (69.0%)	
Compacted	27 (65.9%)	26 (63.4%)	0.8
Unknown	1	1	
Number of Feeders			> 0.9
Multiple	21 (51.2%)	20 (51.3%)	
Single	20 (48.8%)	19 (48.7%)	
Unknown	1	3	
Number of Draining Veins			0.3
Multiple	8 (24.2%)	4 (13.8%)	
Single	25 (75.8%)	25 (86.2%)	
Unknown	9	13	
Location of Draining Veins			0.054
Both	3 (7.3%)	0 (0.0%)	
Deep	6 (14.6%)	13 (31.7%)	
Superficial	32 (78.0%)	28 (68.3%)	
Unknown	1	1	
Venous Stenosis	3 (9.4%)	4 (12.5%)	> 0.9
Unknown	10	10	
Nidal Aneurysm	6 (14.3%)	5 (11.9%)	0.7
SRS modality			
GKRS	0 (NA%)	32 (76.2%)	
Cyberknife	0 (NA%)	5 (11.9%)	
LINAC	0 (NA%)	5 (11.9%)	
Prescription dose, Gy	NA (NA, NA)	20.0 (19.8, 22.0)	
Length of hospital stay, days	9.0 (4.0,24.0)	0.0 (0.0,0.3)	< 0.001
Radiological follow-up, months	12.0 (6.0, 24.0)	33.0 (22.5, 45.5)	0.001
Clinical follow-up, months	12.0 (6.0, 24.0)	27.3 (17.0, 51.0)	< 0.001

^*1*^Median (IQR); n (%) ^*2*^Wilcoxon rank sum test; Pearson's Chi-squared test; Fisher's exact test

For ruptured AVMs (n = 84), there were no significant differences in age, gender, race, or presenting symptoms across the treatment groups. More patients in the resection group were treated within 24 h of presentation (53.8% vs 23.7%, *p* < 0.001). AVM characteristics were similar across the treatment groups. Duration of radiological (*p* = 0.001) and clinical (*p* < 0.001) follow-up was longer for the SRS group.

### Obliteration rate

**Fig. 2:**
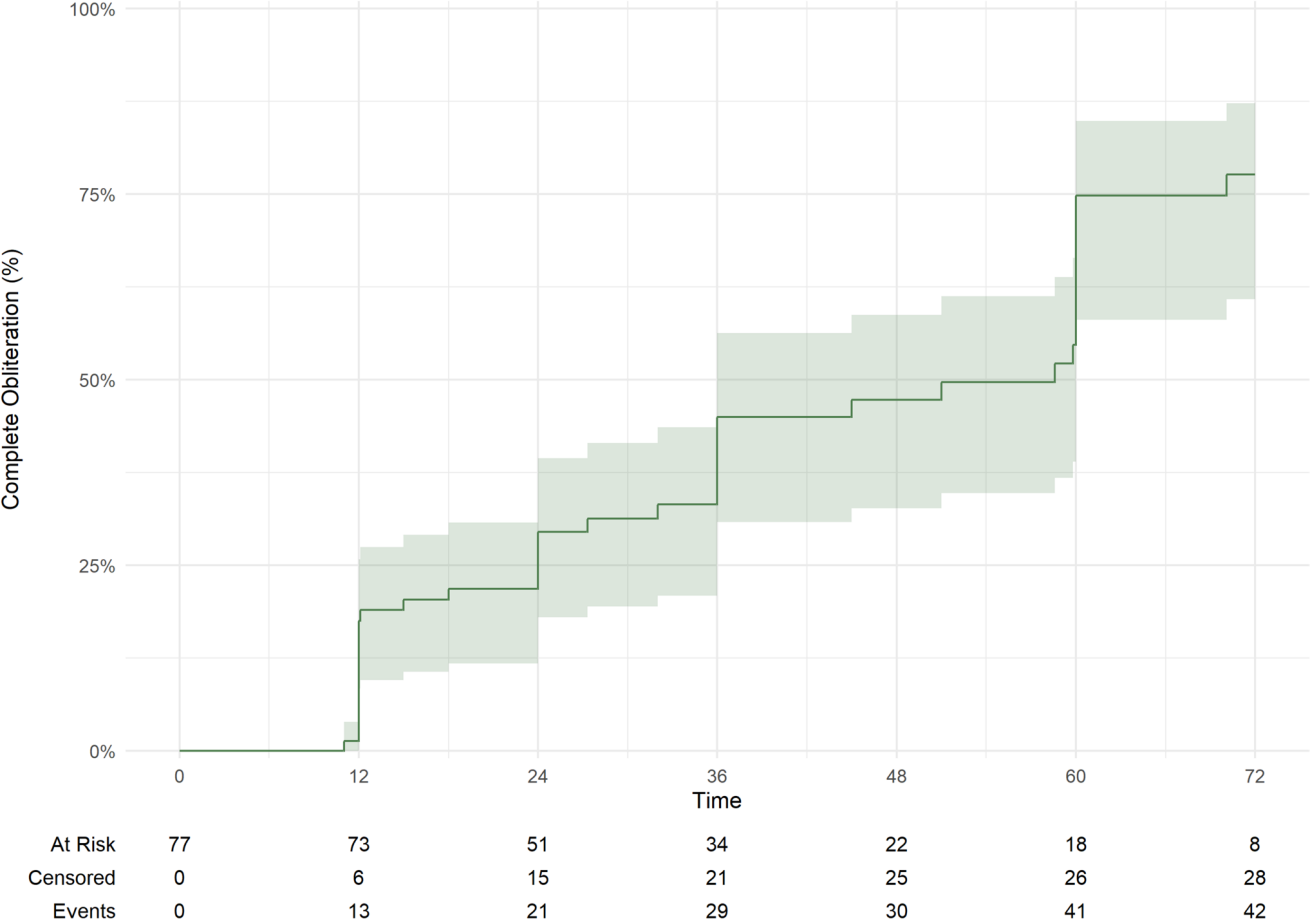
Cumulative incidence of AVM obliteration post-radiosurgery over time

**Fig. 3:**
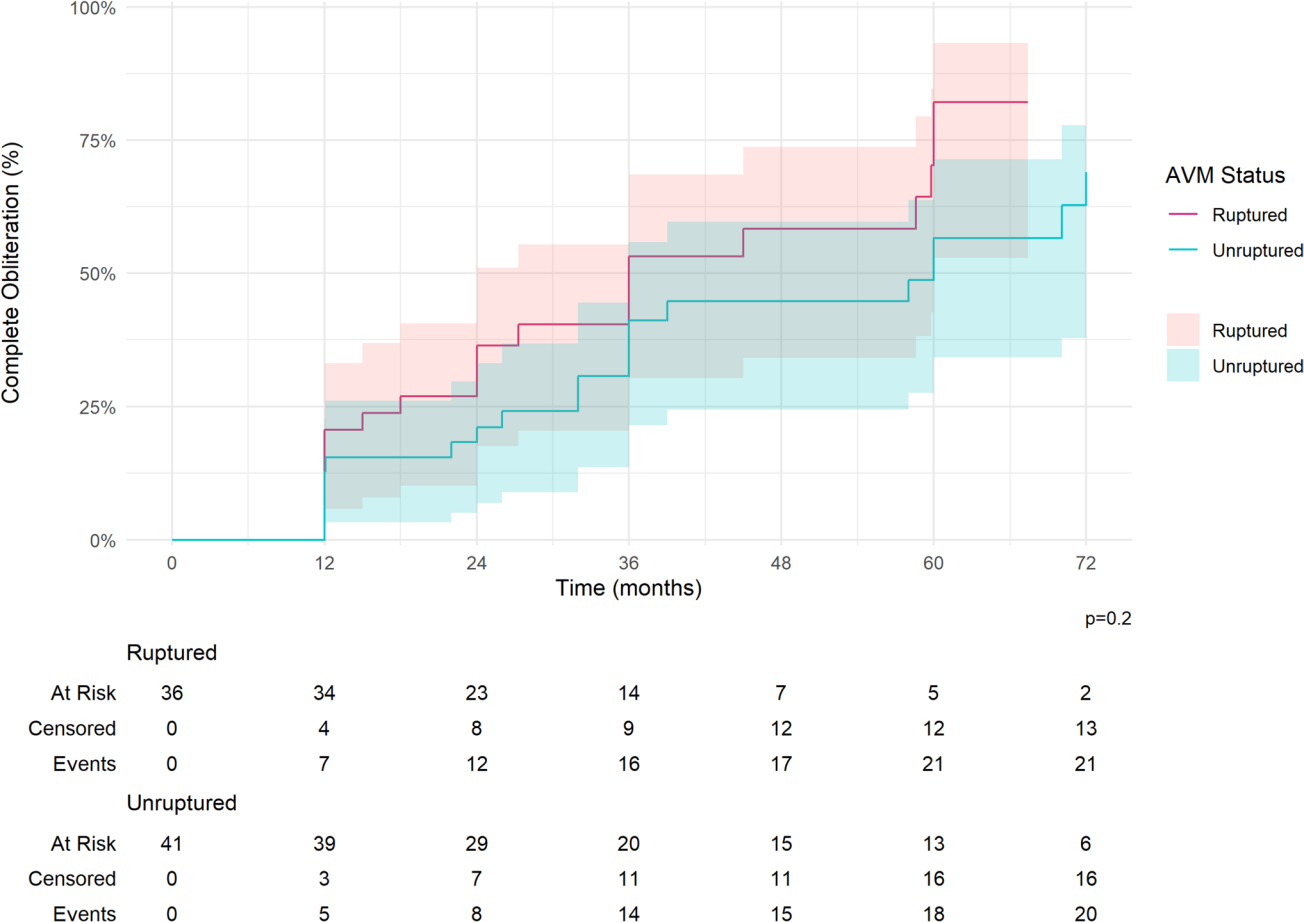
Cumulative incidence of unruptured and unruptured AVM obliteration post-radiosurgery over time

The overall complete obliteration rates for all low-grade AVMs after resection (97.8%) and SRS (60.0%) were significantly different (p < 0.001). The overall median time to obliteration for SRS was 27.3 months (IQR: 12.1, 58.6 months) **(**Fig. 2**, **Table 4**)**. Unruptured AVM outcomes are displayed in Table 5. Resection showed superior obliteration rates compared to SRS (100% vs 58.3%, *p* < 0.001) (Fig. 3**)**. The median time to obliteration for SRS was 32.0 months (IQR: 21.0, 60.0). For ruptured AVMs, resection showed a higher obliteration rate compared with SRS (95.2% vs 61.9%, *p* < 0.001) (Fig. 3). The median time to obliteration after SRS was 24.0 months (IQR: 12.0, 42.0) **(**Tables 6**)**. Radiological follow-up showed that complete obliteration rates after SRS gradually increased over time for overall, unruptured, and ruptured cases (log-rank test, *p* = 0.2), as presented in Table 7.

**Table 4:** Comparisons of outcomes between the matched resection and stereotactic radiosurgery cohorts

Characteristic	Resection, N = 90^*1*^	Radiosurgery, N = 90^*1*^	*p*-value^*2*^
Complete Obliteration	88 (97.8%)	54 (60.0%)	< 0.001
Time to Obliteration, months	NA	27.3 (12.1,58.6)	
Overall Complications	15 (16.7%)	19 (21.1%)	0.4
Symptomatic Complications	10 (11.1%)	10 (11.1%)	> 0.9
Permanent Complications	6 (6.7%)	5 (5.6%)	0.8
Intraoperative Rupture	1 (1.1%)	0 (NA%)	> 0.9
Postoperative Rupture	3 (3.3%)	5 (5.5%)	0.47
mRS at last clinical follow up			0.023
0	42 (52.5%)	60 (70.6%)	
1	24 (30.0%)	9 (10.6%)	
2	6 (7.5%)	7 (8.2%)	
3	3 (3.8%)	4 (4.7%)	
4	2 (2.5%)	0 (0.0%)	
5	0 (0.0%)	1 (1.2%)	
6	3 (3.8%)	4 (4.7%)	
Unknown	10	5	
mRS last 0–2	72 (90.0%)	76 (89.4%)	> 0.9
Unknown	10	5	
Last mRS vs Presentation mRS			
Better*	41/61 (67.2%)	30/45 (66.7%)	0.95
Same	31/80 (38.8%)	45/84 (53.6%)	0.057
Worse	8/80 (10.0%)	9/84 (10.7%)	0.88
Unknown	10	6	
Excellent mRS^+^	14/19 (73.7%)	36/39 (92.3%)	0.053
Unknown	2	2	
All-cause mortality	3 (3.3%)	3 (3.3%)	> 0.9
AVM-related mortality	1 (1.1%)	1 (1.1%)	> 0.9

^*1*^n (%); Median (25%,75%) ^*2*^Pearson's Chi-squared test; Wilcoxon rank sum test; Fisher's exact test^*^After excluding patients who had an mRS score of 0 at presentation, as well as those with an unknown mRS at the last follow-up (unknown: 8 patients in the resection group and 4 in the SRS group — data not shown in the table), the remaining patients included 61 in the resection group (90 – 21 – 8) and 45 in the radiosurgery group (90 – 41 – 4)^#^Number of patients with an mRS score of 0 at the last clinical follow-up among those who had an mRS score of 0 at presentation

**Table 5:** Comparisons of outcomes between the matched resection and stereotactic radiosurgery cohorts for unruptured AVMs

Characteristic	Resection, N = 48^*1*^	Radiosurgery, N = 48^*1*^	*p*-value^*2*^
Complete Obliteration	48 (100.0%)	28 (58.3%)	< 0.001
Time to Obliteration, months	NA (NA, NA)	32.0 (21.0,60.0)	
Overall complications	6 (12.5%)	11 (22.9%)	0.2
Symptomatic Complications	5 (10.4%)	5 (10.4%)	> 0.9
Permanent Complications	2 (4.2%)	3 (6.3%)	> 0.9
Intraoperative Rupture	3 (4.2%)	0 (NA%)	> 0.9
Postoperative Rupture	1 (2.1%)	2 (4.2%)	0.55
mRS at last clinical follow up			0.001
0	22 (55.0%)	37 (80.4%)	
1	16 (40.0%)	4 (8.7%)	
2	1 (2.5%)	3 (6.5%)	
3	0 (0.0%)	1 (2.2%)	
4	1 (2.5%)	0 (0.0%)	
6	0 (0.0%)	1 (2.2%)	
Unknown	8	2	
mRS last 0–2	39 (97.5%)	44 (95.7%)	> 0.9
Unknown	8	2	
Last mRS vs Presentation mRS			
Better	16/29 (55.2%)	10/18 (55.6%)	> 0.9
Same	22/40 (55.0%)	33/46 (71.7%)	0.1
Worse	2/40 (5.0%)	3/46 (6.5%)	0.7
Unknown	8	2	
Excellent mRS^#^	9/11 (81.8%)	27/28 (96.4%)	0.12
Unknown	2	2	
All-cause mortality	1 (2.1%)	0 (0.0%)	> 0.9
AVM-related mortality	0 (0.0%)	0 (NA%)	> 0.9

^*1*^n (%); Median (25%,75%) ^*2*^Pearson's Chi-squared test; Wilcoxon rank sum test; Fisher's exact test ^*^After excluding patients who had an mRS score of 0 at presentation, as well as those with an unknown mRS at the last follow-up (unknown: 6 patients in the resection group and 0 in the SRS group — data not shown in the table), the remaining patients included 29 in the resection group (48 – 13 – 6) and 18 in the radiosurgery group (48 – 30 – 0) ^#^Number of patients with an mRS score of 0 at the last clinical follow-up among those who had an mRS score of 0 at presentation

**Table 6:** Comparisons of outcomes between the matched resection and stereotactic radiosurgery cohorts for ruptured AVMs

Characteristic	Resection, N = 42^*1*^	Radiosurgery, N = 42^*1*^	*p*-value^*2*^
Complete Obliteration	40 (95.2%)	26 (61.9%)	< 0.001
Time to Obliteration, months	NA	24.0 (12.0,42.0)	
Overall complications	9 (21.4%)	8 (19.0%)	0.8
Symptomatic Complications	5 (11.9%)	5 (11.9%)	> 0.9
Permanent Complications	4 (9.5%)	2 (4.8%)	0.7
Intraoperative Rupture	0 (0.0%)	0 (0.0%)	> 0.9
Postoperative Rupture	2 (4.8%)	3 (7.1%)	0.65
mRS at last clinical follow up			> 0.9
0	20 (50.0%)	23 (59.0%)	
1	8 (20.0%)	5 (12.8%)	
2	5 (12.5%)	4 (10.3%)	
3	3 (7.5%)	3 (7.7%)	
4	1 (2.5%)	0 (0.0%)	
5	0 (0.0%)	1 (2.6%)	
6	3 (7.5%)	3 (7.7%)	
Unknown	2	3	
mRS last 0–2	33 (82.5%)	32 (82.1%)	> 0.9
Unknown	2	3	
Last mRS vs Presentation mRS			
Better*	25/32 (78.1%)	20/27 (74.1%)	0.7
Same	9/40 (22.5%)	12/38 (31.6%)	0.36
Worse	6/40 (15.0%)	6/38 (15.8%)	> 0.9
Unknown	2	4	
Excellent mRS^#^	5/8 (62.5%)	9/11 (81.8%)	
All-cause mortality	2 (4.8%)	3 (7.1%)	> 0.9
AVM-related mortality	1 (2.4%)	1 (2.4%)	> 0.9

^*1*^n (%); Median (25%,75%) ^*2*^Pearson's Chi-squared test; Wilcoxon rank sum test; Fisher's exact test ^*^After excluding patients who had an mRS score of 0 at presentation, as well as those with an unknown mRS at the last follow-up (unknown: 2 patients in the resection group and 4 in the SRS group — data not shown in the table), the remaining patients included 32 in the resection group (42 – 8 – 2) and 27 in the radiosurgery group (42 – 11 – 4) ^#^Number of patients with an mRS score of 0 at the last clinical follow-up among those who had an mRS score of 0 at presentation

**Table 7:** Obliteration rate post-SRS on radiological follow-up for overall, unruptured, and ruptured low-grade AVMs

Characteristic	12 months	24 months	36 months	48 months	60 months	72 months	84 months
Overall	18% (8.4%, 26%)	30% (18%, 39%)	45% (31%, 56%)	47% (33%, 59%)	75% (58%, 85%)	78% (61%, 87%)	85% (67%, 93%)
AVM status at presentation							
Ruptured	21% (5.8%, 33%)	36% (18%, 51%)	53% (30%, 69%)	58% (34%, 74%)	82% (53%, 93%)	82% (53%, 93%)	82% (53%, 93%)
Unruptured	13% (1.7%, 23%)	21% (6.9%, 33%)	41% (21%, 56%)	45% (24%, 60%)	57% (34%, 71%)	69% (42%, 83%)	81% (52%, 93%)

### Surgical complications

The overall complication rates, including both symptomatic and asymptomatic cases, were similar for resection (16.7%) and SRS (21.1%, *p* = 0.4) (Table 4). Symptomatic complications were similar for resection and SRS (11.1% each, *p* > 0.9). The rate of permanent complications was not statistically different in the resection group (6.7%) as compared to the SRS group (5.6%, *p* = 0.8). Intraoperative rupture occurred in 1 (1.1%) case in the resection group, while no intraoperative ruptures were reported in the SRS group. Postoperative rupture was observed in 3 (3.3%) cases in the resection group and 5 (5.5%) cases in the SRS group (p = 0.47). For unruptured AVMs, overall complications were higher in SRS (22.9% vs 12.5%, *p* = 0.2), with identical symptomatic rates (10.4%) (Table 5). Permanent complications were slightly more in SRS (6.3% vs. 4.2%, *p* > 0.9). Intraoperative rupture occurred in 3 (4.2%) cases in the resection group for unruptured AVMs, while no intraoperative ruptures were reported in the SRS group. Postoperative rupture was observed in 1 (2.1%) case in the resection group and 2 (4.2%) cases in the SRS group (p = 0.55). In ruptured AVMs, complications were higher in resection (21.4% vs. 19.0%, *p* = 0.8). Symptomatic complications were similar for resection and SRS (11.9% each, *p* > 0.9) with permanent rates higher for resection (9.5% vs 4.8%, p = 0.7) (Table 6). Postoperative rupture was observed in 2 (4.8%) cases in the resection group and 3 (7.1%) cases in the SRS group (p = 0.65).

### Functional outcomes

Functional outcomes generally favored resection regarding changes in mRS scores from presentation to follow-up, with 67.2% of resection patients showing improvement versus 66.7% for SRS (*p* = 0.95) (Table 4). However, 53.6% of SRS patients had unchanged scores compared to 38.8% in the resection group (*p* = 0.057). Worsening scores were similar (10.0% resection vs. 10.7% SRS, *p* = 0.88). All-cause mortality was the same at 3.3% in both groups. Similarly, AVM-related mortality was 1.1% in each treatment group (*p* > 0.9). For unruptured AVMs, 55.2% of resection patients improved versus 55.6% for SRS (*p* > 0.9) (Table 5), and more SRS patients had unchanged scores (55.0% vs. 71.7%, *p* = 0.1). For ruptured AVMs, improvement was higher in resection (78.1% vs. 74.1%, *p* = 0.7), while unchanged scores were more common in SRS (31.6% vs. 22.5%, *p* = 0.36) (Table 6).

## Discussion

**Fig. 4:**
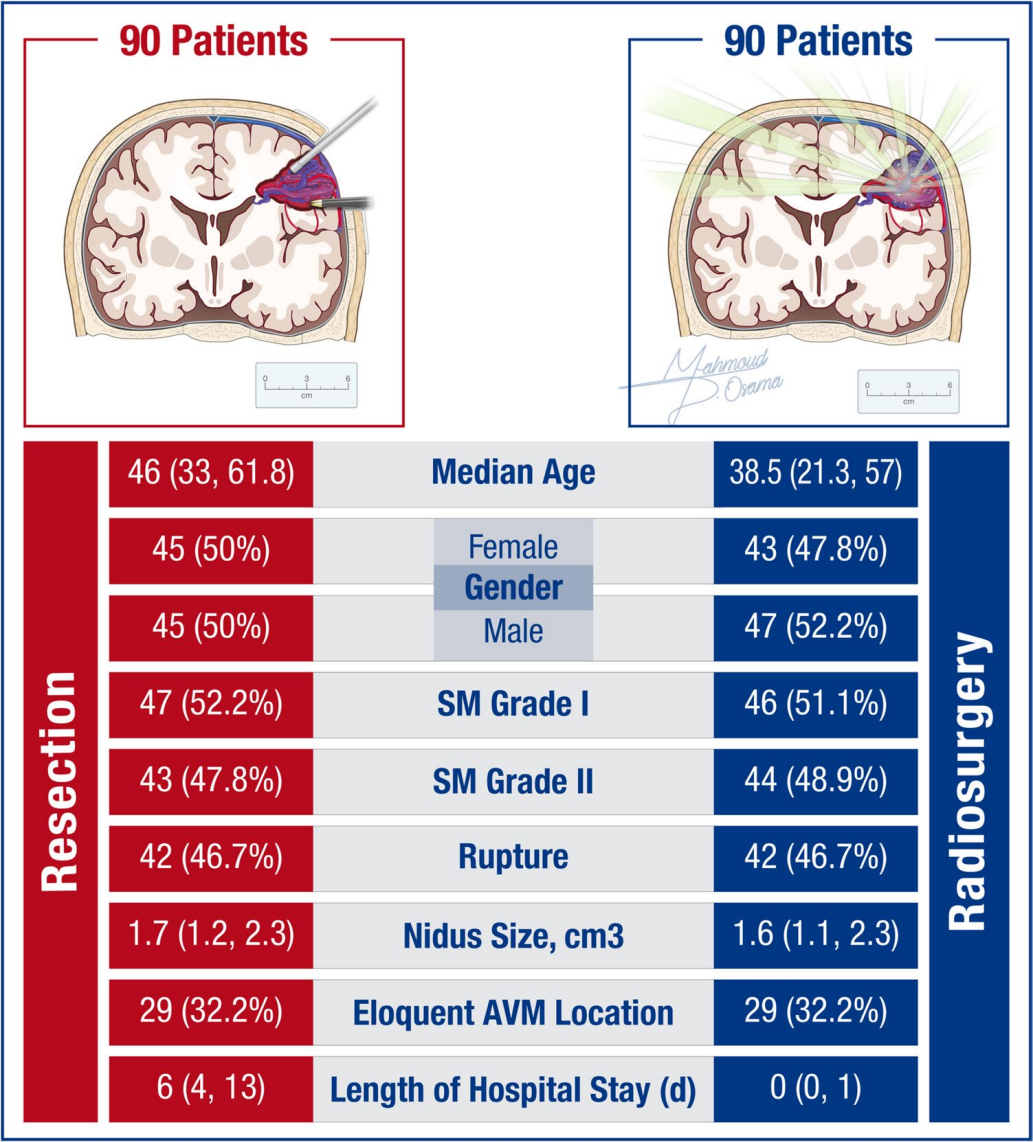
Comparison of the Patient, AVM, and Treatment Characteristics of the Matched Resection and Stereotactic Radiosurgery Cohorts

Our study provides a comparative analysis of resection versus SRS for SM grade I and II cerebral AVMs **(**Fig. 4**).** The primary aim was to evaluate the efficacy and safety of these two treatment modalities based on obliteration rates, complication rates, and functional outcomes in a large multi-institutional consortium.

Our findings demonstrate that resection leads to significantly higher obliteration rates compared to SRS (97.8% vs 60.0%). This result is consistent with previous literature, which has also shown that resection typically results in higher complete obliteration rates for low-grade AVMs.[12–14] A systematic review by van Beijnum et al. highlighted the superior obliteration rates of resection compared to SRS, with resection achieving obliteration at last follow up in approximately 96% of cases versus 38% for SRS.[15] These findings underscore the efficacy of resection in achieving durable AVM obliteration and preventing rebleeding. However, the higher complication rates associated with resection, including new-onset neurological deficits, have prompted ongoing debates regarding the comparative safety of these treatment modalities.[8, 16] The higher obliteration rate observed in our study underscores the efficacy of resection in completely eliminating the nidus of the AVM, thereby reducing the risk of future hemorrhage.

Despite the higher obliteration rates associated with resection, the complication rates remain a critical consideration.[17] In our study, the symptomatic complication rate were similar for resection and SRS (11.1% each, p > 0.9). Permanent complications were slightly higher in the resection group (6.7%) compared to the SRS group (5.6%), but the difference was not statistically significant (*p* = 0.8). These findings align with previous reports, where resection, while effective, often carries a higher risk of complications due to the invasive nature of the procedure.[15]

In clinical practice, AVMs in eloquent brain regions are more frequently treated with SRS due to the higher surgical risks in these critical areas.[6, 18] By balancing our cohort for eloquent AVM location, we may not fully reflect this real-world preference, leading to a selection bias. The literature emphasizes the role of SRS in managing AVMs located in eloquent or deep-seated regions of the brain, where surgical risks are prohibitive. Ding et al. demonstrated favorable outcomes with SRS, particularly for small to medium-sized AVMs, with a median obliteration rate of 76% at 40 months and acceptable complication rates. Maruyama et al. reported that SRS provides a viable treatment option for patients with higher surgical risks, offering a balance between efficacy and safety.[19] Despite these advantages, the delayed obliteration and the risk of hemorrhage during the latency period remain critical challenges in the SRS treatment paradigm.

Our study observed a lower intraoperative rupture rate in the SRS group, with no intraoperative ruptures reported, compared to a 1.1% intraoperative rupture rate in the resection group, though it was not statistically significant (*p* > 0.9). However, postoperative rupture rates were higher in the SRS group compared to the resection group (5.5% vs 3.3%), though it was also not statistically significant (*p* = 0.47). It is important to note that the study may not be sufficiently powered to detect the annual rupture rate typically associated with SRS-treated AVMs. Additionally, the occurrence of post-surgical ruptures in the resection group suggests the possibility of incomplete resection in some cases, which highlights the necessity of angiographic confirmation to ensure complete AVM obliteration. Moreover, it is also important to note that the long-term risk of hemorrhage remains a concern with SRS, particularly during the latency period before obliteration is achieved. Previous studies have highlighted the risk of hemorrhage during this period, emphasizing the need for close monitoring and follow-up.[10]

Functional outcomes change generally favored resection in our study. This suggests that resection not only provides higher immediate obliteration rates but also results in better functional recovery for patients. However, 53.6% of SRS patients had unchanged mRS scores at last follow-up compared to 38.8% in the resection group (*p* = 0.057), indicating that SRS may be associated with more stable functional outcomes without significant deterioration. In patients with an mRS score of 0 at presentation, a higher proportion in the SRS group maintained an excellent outcome (92.3% vs. 73.7%, p = 0.053). Although not statistically significant, this trend suggests a potential preference for SRS in preserving baseline functional status in this subset of patients. The rates of worsening mRS scores were similar between the two groups, underscoring the need to balance the benefits of complete obliteration with the potential risks of functional impairment.

All-cause mortality was the same at 3.3% in both groups. Similarly, AVM-related mortality was 1.1% in each treatment group (*p* > 0.9). These findings are in line with previous reports that documented low and comparable mortality rates for both treatment modalities.[12, 20, 21] The slightly higher AVM-related mortality in the SRS group may be attributed to the risk of hemorrhage during the latency period, as discussed earlier.[22]

Our study highlights key considerations in treatment selection. While resection achieves higher obliteration rates and better functional outcomes, it carries a higher risk of symptomatic complications. Conversely, SRS offers a safer profile with fewer complications but lower obliteration rates. Treatment decisions should be tailored to patient-specific factors such as AVM location, size, and overall health. Resection may be ideal for younger patients with small, superficial, and ruptured AVMs, whereas SRS may be better suited for older patients, those with deep-seated AVMs, or unruptured AVMs at higher risk for surgical complications.

### Study limitations

The retrospective design may introduce selection bias, and despite propensity score matching, unmeasured confounders could influence results. Variability in follow-up duration, particularly longer observation for SRS patients, may affect complication detection and obliteration rates. Multicenter data introduced heterogeneity in treatment protocols, complicating standardization. Excluding patients with incomplete records may limit the generalizability, as these criteria do not fully reflect the diverse clinical population. Furthermore, excluding patients who received prior treatments, such as endovascular interventions, may also impact the generalizability of the findings and influence SRS outcomes, particularly in Grade II AVMs with larger diameters or in critical locations. However, including patients with prior treatments may have introduced biases that could not be easily recognized and controlled for in this study. The mRS may not capture subtle neurological or quality-of-life changes. Additionally, the small sample size, particularly in subgroup analyses, limits the detection of rare complications or long-term outcomes. A further limitation of this study is the potential for selection bias, as AVMs with favorable characteristics are more likely to undergo resection, while higher-risk AVMs are often referred to SRS, potentially influencing obliteration rates. Additionally, the Spetzler-Martin grading system, originally designed for surgery, has limitations in predicting SRS outcomes, particularly in its assessment of AVM size and eloquent locations, which may impact its applicability to radiosurgical decision-making. Further research is needed to develop grading systems specifically tailored to SRS, incorporating factors such as AVM angioarchitecture, patient characteristics, and radiosurgical response to improve outcome prediction and treatment planning. Finally, as the median follow-up of 36 months may not fully capture the latency period of SRS, longer follow-up studies are needed to better assess its long-term efficacy.

## Conclusion

Both microsurgical resection and SRS are effective treatments for Spetzler-Martin grade I and II cerebral AVMs. Our study found that while both modalities offer favorable outcomes, resection is associated with immediate obliteration, whereas SRS offers a less invasive option with a delayed effect. These findings support the importance of individualized treatment planning based on specific patient characteristics and AVM features.

## Data Availability

No datasets were generated or analysed during the current study.

## Abbreviations

MISTA: Multicenter International Study for Treatment of Brain AVMs
AVMs: Arteriovenous malformations
SM: Spetzler-Martin
SRS: Stereotactic radiosurgery
GK: Gamma knife
LINAC: Linear accelerator
mRS: Modified Rankin Scale
PSM: Propensity score matching
STROBE: Strengthening the Reporting of Observational Studies in Epidemiology
MRI: Magnetic Resonance Imaging
MRA: Magnetic resonance angiography
CTA: Computed tomography angiography
